# Self-supervised and semi-supervised learning for road condition estimation from distributed road-side cameras

**DOI:** 10.1038/s41598-022-26180-4

**Published:** 2022-12-26

**Authors:** Fabio Garcea, Giacomo Blanco, Alberto Croci, Fabrizio Lamberti, Riccardo Mamone, Ruben Ricupero, Lia Morra, Paola Allamano

**Affiliations:** 1 Dipartimento di Automatica e Informatica, Politecnico di Torino, C.so Duca degli Abruzzi, 24, 10129 Turin, Italy; 2 Waterview srl, Turin, Italy

**Keywords:** Environmental sciences, Natural hazards, Computer science, Climate change, Hydrology

## Abstract

Monitoring road conditions, e.g., water build-up due to intense rainfall, plays a fundamental role in ensuring road safety while increasing resilience to the effects of climate change. Distributed cameras provide an easy and affordable alternative to instrumented weather stations, enabling diffused and capillary road monitoring. Here, we propose a deep learning-based solution to automatically detect wet road events in continuous video streams acquired by road-side surveillance cameras. Our contribution is two-fold: first, we employ a convolutional Long Short-Term Memory model (convLSTM) to detect subtle changes in the road appearance, introducing a novel temporally consistent data augmentation to increase robustness to outdoor illumination conditions. Second, we present a contrastive self-supervised framework that is uniquely tailored to surveillance camera networks. The proposed technique was validated on a large-scale dataset comprising roughly 2000 full day sequences (roughly 400K video frames, of which 300K unlabelled), acquired from several road-side cameras over a span of two years. Experimental results show the effectiveness of self-supervised and semi-supervised learning, increasing the frame classification performance (measured by the Area under the ROC curve) from 0.86 to 0.92. From the standpoint of event detection, we show that incorporating temporal features through a convLSTM model both improves the detection rate of wet road events (+ 10%) and reduces false positive alarms ($-$ 45%). The proposed techniques could benefit also other tasks related to weather analysis from road-side and vehicle-mounted cameras.

## Introduction

Adverse weather conditions, such as snow and rainfall, have been consistently linked to an increase in the frequency and severity of accidents on roads and highways^1,2^. In particular, slippery road conditions due to the presence of water on the surface are a major contributing factor. While several methods have already been proposed to detect rainfall and snowfall^3,4^, estimating road conditions is a separate, albeit related, problem. Besides the intensity of rainfall, a variety of factors (like, e.g., the type of asphalt, the road morphology, etc.) can alter the speed at which the water builds up and is drained. Depending on the weather conditions, water can persist on the road long after raining has stopped. The availability of accurate and real-time information about road conditions can be beneficial for scheduling road maintenance, providing safety warnings for drivers, and arranging other accident-preventing interventions. In addition, since the frequency of extreme weather events is rapidly increasing due to climate change, collecting such information is crucial to monitor its impact on road infrastructures^5^.

Road Weather Information Systems (RWIS) stations are equipped with a variety of sensors, often including cameras, and can be used to provide real-time road condition and weather information at selected point locations. However, RWIS stations are in limited number due to their installation and operating costs, and this can introduce wide gaps in available data^6^. For this reason, many alternative sensing approaches have been proposed, including on-vehicle cameras^7,8^, outdoor and surveillance cameras^6,9^, acoustic sensors^10–12^, or a combination of images and meteorological sensors^9^. Road-side cameras are particularly attractive as they provide a readily-available, diffuse network of sensors, both at the urban and extra-urban level.

With respect to data analysis, Deep Convolutional Neural Networks (CNNs) have been applied with encouraging results to weather classification^13–15^ and, more recently, road-condition estimation^6^. The vast majority of the proposed techniques perform the classification on a frame-by-frame basis, which achieves good results for well-separated classes (e.g, sunny, cloudy, snowy). However, to detect fine-grained changes in road conditions, it is crucial to account for the temporal correlation between adjacent frames. It is also important to compare algorithms in their ability to detect with precision the start and end time of wet road events, beyond simple by-frame accuracy metrics which do not reflect the expectations and needs of the end users.

In this paper, we propose a semi-supervised learning (SSL) approach to train a convolutional Long Short-Term Memory networks (convLSTM) tailored to the problem of wet road detection. We further provide extensive validation on a real-life dataset which contains continuous video streams spanning several months of acquisition. Thus, we are able to paint a realistic picture of the achieved performance, fully accounting for the whole range of illumination and weather conditions that arise during the year.

Specifically, our contribution is as follows:

- We introduce a semi-supervised training technique that leverages unlabelled data through a combination of novel self-supervised pre-training and established semi-supervised training techniques. Specifically, we tailor contrastive self-supervised pre-training for road-side and surveillance camera video streams;
- We design and train a convLSTM for road condition classification, including novel temporally consistent data augmentation techniques that mimic outdoor illumination changes;
- We evaluate by-frame (CNN) and temporal (convLSTM) models on an extensive and well-balanced dataset that accounts for a wide variety of acquisition, illumination, and weather conditions, defining appropriate per-event metrics that capture the ability to precisely identify the start and end time of wet road events.

The rest of the paper is organized as follows. In Section “Related work”, we provide the most relevant background on weather and road condition estimation, event detection and self-supervised learning. In Section “Dataset description”, we summarize the main characteristics of the dataset used to train and test the models. In Section “Methodology”, we describe the key principles behind the training strategies adopted to solve the task. In Section “Experimental settings”, we report in detail the settings used to carry out the experiments, and in Section “Results”, we summarize the results. Finally, in Sections “Discussion” and “Conclusions and future work”, we discuss our findings and propose several directions for future research.

## Related work

### Weather and road condition estimation

Existing approaches for weather and road condition estimation can be roughly categorized based on the acquisition setting (fixed vs. vehicle-mounted sensors), the sensor(s) used, and the algorithmic approaches (physics-based vs. data-driven). These tasks can be further approached from the viewpoint of automotive applications, which rely on one or more vehicle-mounted sensors^7,8,15^, or from the perspective of road infrastructure management, which relies instead on distributed, fixed sensors^6,13,14^. The present study falls in the latter group, on which we will focus our attention in the remainder of this section.

Traditionally, distributed RWIS stations have been employed to map real-time atmospheric parameters, road pavement conditions and visibility. Such stations can be equipped with a variety of sensors, including thermometers, visibility sensors, rain gauges, surface sensors, and sometimes cameras. Their installation and operating costs, however, limit the number of available stations, resulting in low spatial resolutions.

Outdoor and surveillance cameras provide a ready-to-use, scalable alternative, with minimal installation costs^6,9,16^. However, detecting road conditions from outdoor cameras is challenging, due to the large distance between the camera and the road surface, as well as the wide range of illumination conditions observed in the wild. In addition, frames need to be manually labelled as most cameras are located too far from RWIS stations. For this reason, semi-supervised techniques have been proposed to lower the cost associated to manual annotation^6^.

Both physics- and data-driven methods have been devised for weather and road condition estimation. Several physics-based methods are tailored to the detection of weather-related phenomena, such as rain streaks, fogs or snow^17–20^. For instance, Allamano et al.^17^ proposed a camera-based rain gauge which measures the intensity of rain fall rate from a single camera. After detecting individual rain drops, their speed and diameter are calculated by solving a system of equations that relate their physical properties to the observed image intensities, after compensating for motion blur. Physics-based methods offer greater interpretability and stability over data-driven ones. However, it is impractical to derive a mathematical formulation for complex phenomena, such as water build-up on the road surface. Furthermore, physics-based models are dependent on camera parameters, such as focus distance and time of exposure^17^.

Deep learning-based techniques, such as CNNs, have proven to be a robust alternative in many related applications^6,7,14,15^. Unlike physics-based methods, they do not require calibrated camera sensors, and can be trained to achieve robustness to varying acquisition and illumination conditions, which is essential to effectively leverage existing camera networks. The vast majority of existing works are based on fine-tuning a state-of-the-art CNN to perform a frame-by-frame classification, and do not take into account the temporal correlation between subsequent frames^6–8,13–15^.

The closest related work is the one by Ramanna et al.^6^, who proposed a real-time system for monitoring road conditions. The project leverages 1.5 million snapshots taken by street and highway cameras located across North America at different times of the day, spanning both urban and rural scenarios. A semi-supervised strategy is applied to incrementally label the data and fine-tune standard CNN models towards the task of road condition classification. However, there are several limitations in the work by Ramanna et al.^6^, both in terms of datasets and deep learning model. On the one hand, the dataset was sampled over a period of three months, whereas the proposed dataset spans a period of two years and includes cameras sampled continuously over several months. On the other hand, our study shows that a temporal model, such as a convolutional LSTM, substantially outperforms a frame-by-frame approach, reducing false alarms and capturing more confidently the temporal evolution of the phenomenon under analysis. We propose a complete pipeline for self-supervised, semi-supervised training of this temporal model, leveraging spatially and temporally consistent data augmentation to increase its generalization capability.

### Event detection in video streams

The present work is related to the field of event and action recognition in untrimmed video streams^21^. While many definitions of event have been proposed in literature, we refer in this context to a “wet road event” as a series of consecutive frames labelled as wet. In this respect, the task at hand bears some resemblance with the well-known problem of *temporal action localisation in video streams*, which requires to identify, as precisely as possible, the temporal boundaries of each action/event^22^. We leverage the existing body of literature in this field to draw inspiration for our evaluation protocol.

The problem of wet road detection falls under the umbrella of online event detection, in which the goal is *“to detect an action as it happens and ideally even before the action is fully completed”*^23^, processing new video frames immediately, without resorting to future observations. Possible approaches to temporal modelling for online action detection include combining features extracted by a CNN using a suitable temporal modelling technique, such as temporal pooling, recurrent neural networks, temporal convolutional models or temporal attention models^24^. A recent comparison on the TVSeries^23^ and THUMOS-14 datasets^25^ showed that LSTM can outperform other approaches, although best results could be obtained by combining different models^24^. Likewise, we based our work on a convLSTM model, although we found very similar performance when using a temporal convolutional model.

### Self-supervised learning

We resort to self-supervised pre-training to leverage the availability of non-annotated frames. Self-supervised approaches learn semantically meaningful feature representations via pretext tasks that do not require semantic annotations, but do require higher-level semantic understanding in order to be solved^26^. The resulting representations can be then fine-tuned to the target downstream task using the subset of labelled data. Previous results showed that a combination of self-supervised pre-training and pseudo-labelling outperforms pseudo-labelling alone in a semi-supervised setting^27^.

Our approach is inspired by the family of contrastive self-supervised learning approaches^28,29^, which were shown empirically to be highly effective when transferring to fine-grained image classification^26^. Most existing contrastive self-supervised techniques treat each image as a unique instance, and rely on aggressive data augmentation to generate different views^26,28,30^. Spatio-temporal extensions that were proposed for video sequences likewise assume that each sequence represents a different scene^31^. Neither approaches are suitable for the present dataset, which contains a large number of consecutive frames for a relatively small number of scenes acquired by fixed cameras.

Self-supervised approaches have also been tailored to remote sensing^32,33^. In remote sensing, as in our dataset, multiple images of the same area are acquired at regular time intervals. However, our dataset is sampled sparsely along the spatial dimension and at high resolution along the temporal dimension, whereas satellite imagery is sampled continuously in the spatial dimension, but at sparse time intervals. In addition, some of the proposed techniques are not applicable, as they rely on the multi-spectral nature of satellite imagery^33^; others are designed to encode similarity based on the geographical distance, and promote invariance with respect to location^32^, whereas in our context the network must be sensitive to subtle changes in road appearance.

Instead, our approach is inspired by applications of self-supervised learning in the medical domain^29^, in which similarity between two images is rooted on discrete (or discretized) variables such as lesions type, location and size.

## Dataset description

**Figure 1:**
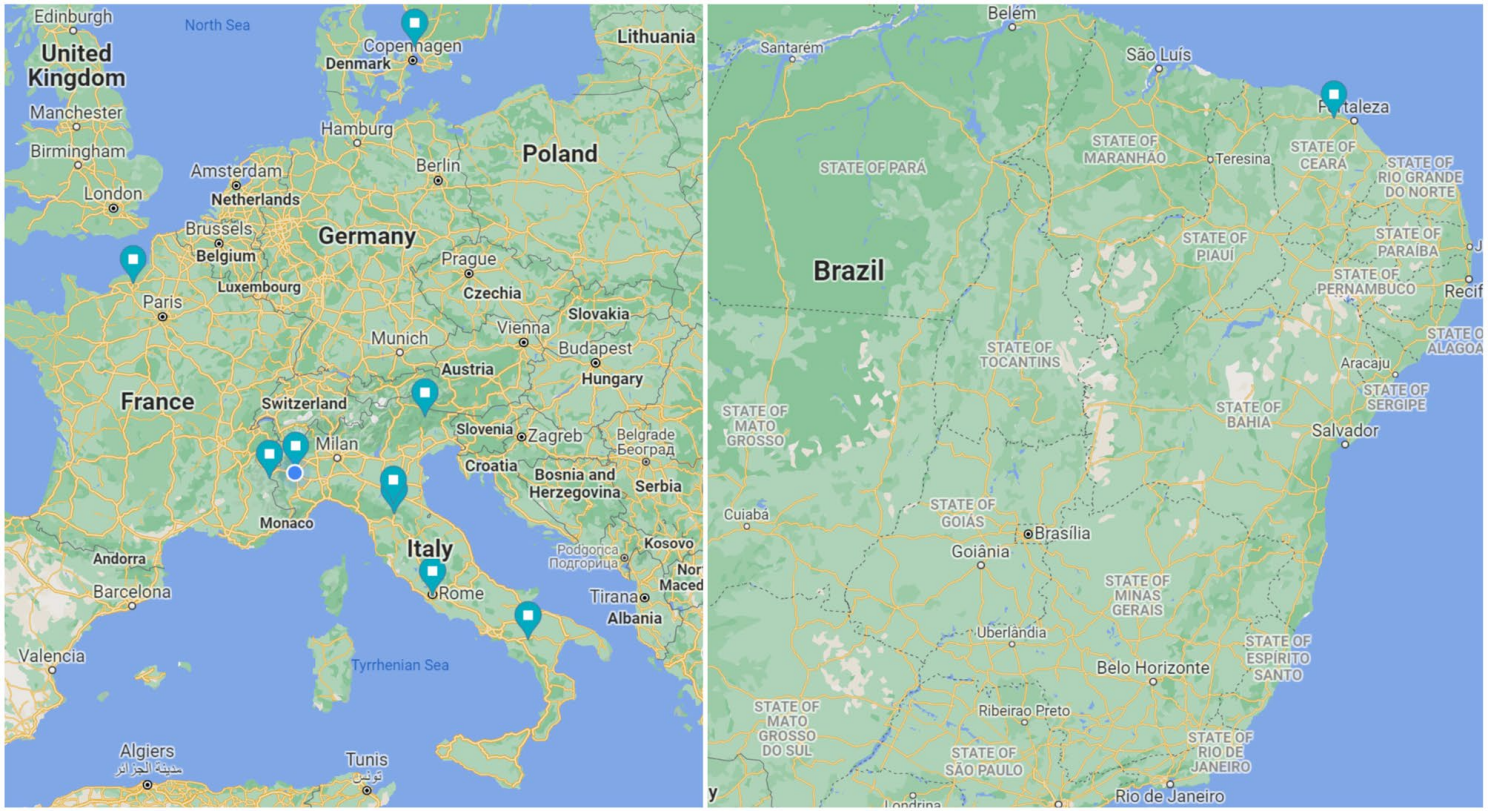
Geographical locations of the cameras included in the analysis.

The present study was conducted on a real-life dataset acquired between July 2018 and January 2021 from 25 roadside Full HD cameras located predominately in the Northern hemisphere (Europe). Most cameras were located in an extra-urban setting, along important segments of the road infrastructure, encompassing a variety of climate, weather, and traffic conditions. The images used in this study were acquired from a mix of bullet and PTZ (Pan-Tilt-Zoom) cameras^34^. All cameras were mounted at a height of roughly 5m and tilted towards the road at an angle of 10$^{\circ }$–20$^{\circ }$. Figure 1 shows the geographical locations of the cameras included in the analysis, and Table 1 the corresponding acquisition periods. Each camera, originally sampled at 12 frames/minute, was further downsampled at 1.3 frames/minute to reduce the computational complexity of the network. Only frames acquired between sunset and sunrise were included. Many cameras were equipped with a light sensor to distinguish between daytime and nighttime operations. In other cases, sunset and sunrise hours were estimated using the ephemeris equations^35^ and geographical information. Additional characteristics of the cameras are reported in the Appendix. In this section, we will first describe the data acquisition and selection process by employing tools and methods from the causality literature (Section “Causal analysis”), illustrate how data was annotated (Section “Annotation process”), and then present the dataset properties and the training/validation split (Section “Dataset distribution and training/validation split”).

**Table 1:** Data acquisition period for each camera site included in the analysis.

Camera site	A1	A2	A3	A4	A5	A6
Acquisition period	02/2020–05/2020	02/2020–05/2020	07/2018–07/2019	10/2018–07/2019	10/2018–05/2020	10/2018–07/2019

Camera site	A7	A8	A9	A10	B1–B15	
Acquisition period	07/2018–09/2018	10/2019–11/2019	08/2018–07/2019	02/2020–05/2020	12/2020–01/2021	

### Causal analysis

Several factors influence road conditions and, thus, need to be taken into account when sampling and stratifying available data in a training and validation set. A causal diagram provides an intuitive, yet rigorous formalism to identify both latent and observed factors at play^36–38^. The main purpose of this analysis was to provide a solid foundation on which data frames could be sampled and split into a training and validation set, as well as to characterize the properties of the domain on which the classifier is trained.

**Figure 2:**
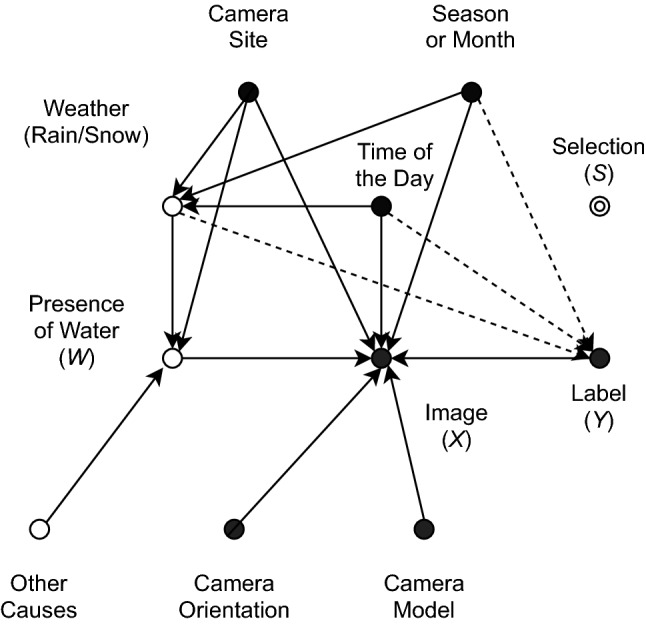
Causal model for the prediction of wet road conditions. Hidden variables are depicted with empty circles.

The causal diagram for the selected task, namely, inferring the presence of water from video frames acquired along a given road stretch, is depicted in Fig. 2. The classification task entails predicting the probability *Y* of water being present on the road given a frame, or sequence of frames, *X*—alternatively, *Y* could represent the label space representing different weather conditions such as *wet*, *dry*, or *snowy*. The actual presence of water is represented by the hidden variable *W*, since in our setting it was not possible to directly observe the phenomenon, e.g., by deploying on-field sensors.

Water builds on the road due to a variety of reasons. Weather conditions are of course decisive, but the interplay between climatic conditions and the road drainage system is also fundamental: in fact, water build-up may not occur, e.g., in the case of light rain, and conversely could persist for a long time after heavy rainfall. In our diagram, weather is assumed to be a hidden variable, as we did not have access to or use weather reports, but of course weather conditions can be largely inferred from the images themselves. Variables like camera site, time of day and season act as confounders, as they affect both illumination conditions and the frequency of water build-ups. The camera site variable embeds multiple aspects, including geographical location (longitude/latitude), road morphology, type of asphalt, road drainage, and so forth. All these factors influence the frequency and characteristics of wet road events. The image *X* also acts as a collider between the true phenomenon *W* and other independent factors, such as camera orientation and model. Hence, care must be taken as the presence of colliders may lead to biased datasets^39,40^.

Based on the devised diagram, the task could be defined *causal*, since the label is inferred directly from the input frame, which acts as a mediator with respect to the underlying phenomenon^41^. On the other hand, the annotators were aware of the season, time of the day and weather, which were either available or could be easily inferred from the image, as represented by the dashed arrows in the diagram. Thus, even though annotators were specifically instructed to not rely on these variables for the classification task (for instance, the road could be classified as dry in the case of rain, if there was no visible water build-up), it is conceivable that this information is useful for human raters to solve the task. Hence, from this standpoint, the task can be also seen as *confounded*, and hence *anti-causal*, which is consistent with theoretical conjectures that SSL should not improve the performance of causal tasks^41^.

### Annotation process

A subset of the data was manually annotated to provide a set of clean labels for validation and testing, as well as an initial seed to which SSL strategies could be applied. In addition, the annotated subset was used to transfer the representation learned by self-supervised learning to the target task. Specifically, sites B1–B15 were fully annotated, since the acquisition period was short, and many sites included very challenging illumination conditions that were not adequately represented in sites A1–A10. For sites A1–A10, a predetermined number of days per site was randomly selected to be annotated.

The annotation process involved five raters: two expert and three junior raters. Expert raters set the criteria for labelling and provided the initial training. Consensus meetings were used to review initial annotations, as well as to resolve unclear or borderline cases. A frame was labelled as wet when the amount of water build-up and/or the presence of water pools were deemed sufficient to reduce vehicles traction and/or increase the risk of accidents in general. All other frames were labelled as dry, even if the road was moist or drying up. This choice is potentially more challenging than in previous works, in which the wet class included “a spectrum of conditions from moist roads to puddles to soaking wet”^6^. However, a very broad definition of wet road would lead to an unacceptable false positive rate in production environments.

Even though the problem was originally cast as a binary classification problem, auxiliary classes were defined to account for all cases in which it was not possible to assign a label:

- *Poor visibility*: this class represents cases in which fog or other weather conditions reduce visibility to the point that it is not possible to assign a confident label;
- *Dark*: this class represents frames in which the road is severely under-exposed or under-illuminated: this condition typically occurs when the start or the end of the day is not identified with precision;
- *Offline*: this class represents the static feed when the camera goes offline, e.g., due to network errors;
- *Over-exposed*: this class represents cases in which the frame is too over-exposed to clearly assess the state of the road: this condition typically occurs around mid-day, depending on how the camera is oriented.

To reduce the time burden, only a small portion of the dataset (correspondingly to roughly 3000 frames) was completely annotated by hand. For the remaining frames, a classifier was trained to produce tentative labels which were then manually refined, following a process similar to Ramanna et al.^6^. Specifically, features were extracted by fine-tuning a ResNet50 CNN pre-trained on ImageNet, and then applying a k-Nearest Neighbour classifier ($k=50$). The proposed labels were grouped by camera and denoised using a majority filter to exploit temporal consistency. Then, all labels were manually refined and confirmed using the Microsoft CVAT tool^42^, in which each day of acquisition was loaded as a separate video. Critical or borderline cases were discussed in consensus meetings, as previously outlined. Hence, we assume this process to be equivalent to manual annotation and, thus, reasonably noise-free.

All frames labelled as poor visibility, dark, offline or over-exposed were removed from the resulting dataset. It is worth noticing that these special classes were used only when manually labelling the data, but were not included as additional classes due to their low prevalence, as it will be discussed in Section “CNN model and training”. The sole exception were over-exposed frames, which were automatically discarded by applying a threshold on the average intensity; this simple trick proved equivalent to manually labelling on the annotated subset.

### Dataset distribution and training/validation split

The overall dataset contains roughly 400,000 frames acquired from 24 cameras. The annotated subset was split into a training and validation set, and the rest was kept as additional training data.

The dataset was sampled by considering the day as the atomic unit. Since night frames were excluded from the analysis, each day constituted a disjoint and non-contiguous set of frames. All the frames acquired in a day from a single camera are referred to as a *camera day* in the following. Hence, each camera day was either assigned to the training set or the validation set and was either annotated or not. This strategy ensures that the frames in the validation and testing sets are not statistically correlated, thus inflating performance, and allows to evaluate the performance on a per-event, rather than per-frame basis. In addition, all illumination conditions that arise during the day are equally represented, avoiding biases.

The training/validation/test split was performed at the camera day level taking into account the main confounders discussed in Section “Causal analysis”, especially the camera site and the season.

As reported in Table 1, the available number of camera days varied across sites. Sites with more than 50 camera days (6) were randomly split into a training and validation-test set, to ensure that all seasons were equally represented. The validation-test set was further split into a validation (50%) and test set (50%), stratifying by camera orientation. Sites with less than 10 camera days (15) were either assigned to the training or validation set: for these sites, it was not feasible to sequester part of the data for external testing. Sites were manually assigned to ensure that sites with similar characteristics and morphology (presence of elevated roads, bridges, or tunnels) were evenly split across the two datasets. This strategy guarantees that the training set and the validation set encompass similar acquisition conditions, while ensuring that the network is not memorizing each acquisition site. Additionally, sites outside of the Northern hemisphere were included in the training set, as the amount of data available would be insufficient to test the performance of the model in these climatic conditions.

The resulting distribution of the training and validation sets is reported in Table 2, in which Training-50K refers to the annotated portion of the training set, whereas Training-300K refers to the complete set which includes both annotated and non-annotated frames.

Finally, to account for the temporal continuity, we defined an *event* as a sequence of consecutive frames labelled as wet. This definition will be further discussed in Section “Performance assessment” in relation to the performance assessment. As illustrated in Table 2, very short events (less than 15 min) and very long events (more than 6 h) are infrequent, and many wet events start in the morning, before 9am.

**Table 2:** Distribution of the training, validation and testing sets.

	Training-300K	Training-50K	Validation Set	Test Set
Total frames	307,034	50,152	47,613	42,164
Dry frames	245,087	43,038	41,837	38,786
Wet frames	61,947	7114	5776	3378
Camera sites	14	14	16	9
Camera days	1458	263	239	208
Wet events	–	172	118	78
Wet events longer than 120 frames (360 min)	–	12	13	12
Wet events shorter than 5 frames (15 min)	–	23	14	8
Wet events starting in the morning (before 9AM)	–	58	52	34

## Methodology

In this section we describe the proposed approach for wet road detection. First, the techniques employed for data preprocessing and data augmentation are presented (Section “Data preprocessing and data augmentation”). The proposed approach is based on training models of incremental complexity. First, a CNN is trained exploiting self-supervised and SSL techniques (Section “CNN model and training”). Then, the feature extractor is incorporated into a temporal convLSTM model, which is further fine-tuned on video sequences (Section “Temporal (convLSTM) model and training”).

### Data preprocessing and data augmentation

Many surveillance camera feeds include a text overlay reporting information such as acquisition timestamp and/or location. All images are thus preprocessed to remove text overlays: for this operation, simple thresholding and morphological operators were sufficient as the text is usually either black or white.

Two different approaches have been adopted to perform data augmentation for the CNN and convLSTM models. For the former, the following transformations were randomly applied independently to each frame: shear (in the range $-$ 45 to + 45 degrees), horizontal flipping (20%), cropping (in the range $-$ 0.05% to 0.10%), perspective transformation (0.1 ratio), random rotation (in the range $-$ 45 to + 45 degrees), additive Gaussian noise, coarse dropout (3–15% of the pixels), and brightness variation (in the range $-$ 30 to + 30).

**Figure 3:**
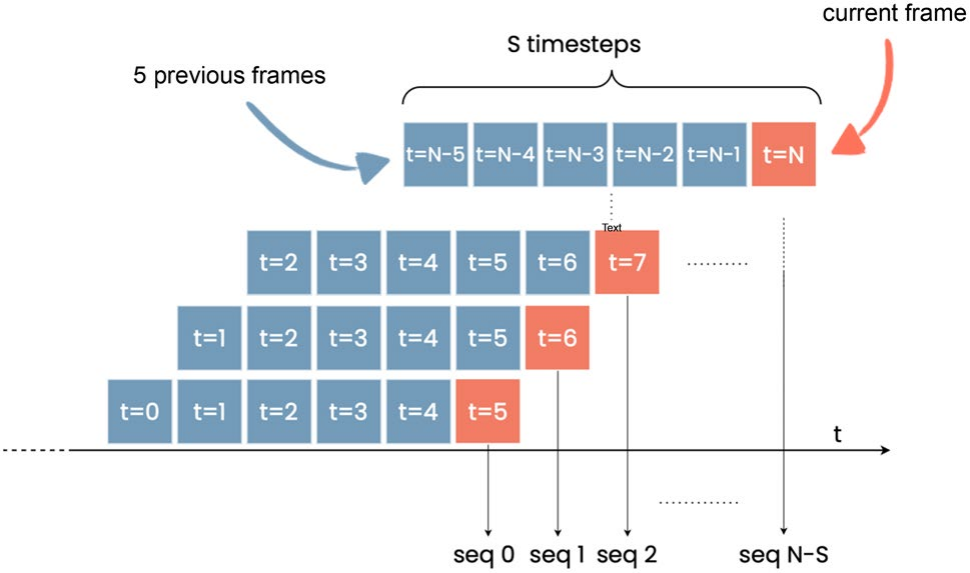
Sequence generation. The sequence generated for each frame included the $S-1$ previous frames.

The temporal convLSTM model was, in turn, trained and tested on a sequence of *N* consecutive frames, as shown in Fig. 3. Hence, data augmentation should generate temporally and spatially consistent sequences, allowing the network to capture subtle changes in the appearance of the road. For spatial and geometric transformations, we adopted the temporally consistent method proposed in Qian et al.^31^: a random set of transformations (shear, flipping, cropping, perspective, rotation, additive noise, and dropout) is sampled for each sequence and applied to all frames consistently. This approach imposes strong spatial augmentation on each frame without breaking the temporal consistency across the sequence. It is also consistent with the real data points, which are acquired from fixed cameras. Settings are the same as in the CNN model.

**Figure 4:**
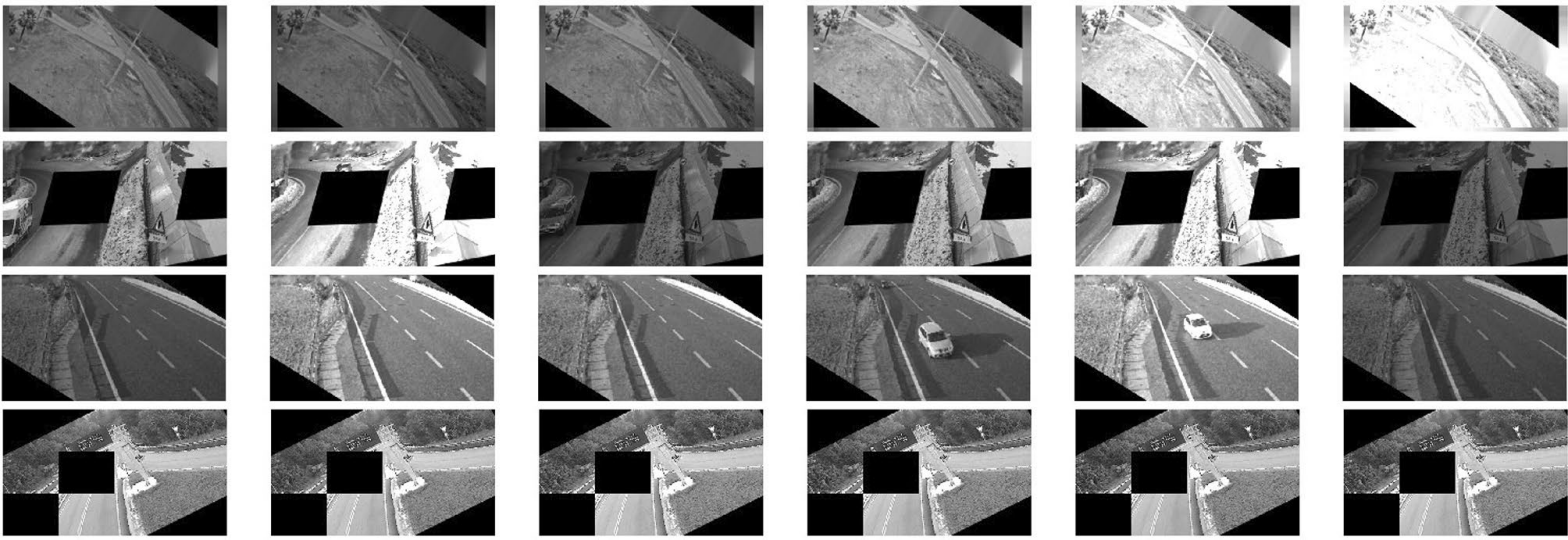
Examples of artificial sequences generated by combining temporally coherent spatial augmentation and simulated outdoor illumination conditions.

For brightness, we introduced a novel transformation which simulates changes in illumination conditions in outdoor scenes. Such changes can be both slow, due to the shifting position of the sun with respect to the camera, and fast, due to the shadows of rapidly moving clouds. Depending on the orientation of the camera (North, South, East, or West), as well as its positioning with respect to the line of the horizon, these illumination changes may result in temporary under- or over-exposure. Rapid changes in exposure, which cause the road appearance to darken, may mimic water build-up and, thus, increase the false positive rate. To make the network more robust to these natural phenomena, we multiplied the intensity of the *i-*th frame by a factor $A\sin (k(i+b))$, with randomly selected amplitude *A*, period $\frac{2\pi }{k}$ and phase *b*. The parameters were set to have a maximum brightness increase of 2 (up to double the original brightness) and a maximum decrease of 0.5 (down to half of the original brightness). Depending on the parameters, this simple transformation can simulate a wide range of different illumination and climatic conditions, from almost constant illumination to very rapid shifts. Examples of augmented video sequences are shown in Fig. 4.

### CNN model and training

#### Baseline

The chosen *baseline* model was a Resnet50 network pre-trained on ImageNet^43^. Since wet road events are relatively rare, and class imbalance could negatively impact both the speed of convergence and the generalization of the model on the test set^44^, the *focal loss* function was chosen for all experiments. Oversampling by repetition, a common strategy to reduce the impact of data imbalance, was found prone to overfitting.

For the loss, we adopted the multi-class formulation proposed by Cui et al.^45^. Shortly, this framework associates to each sample a small neighborhood region, instead of a single point. To this aim, the *effective number* of samples $E_{n_{y}}$ is calculated as $\left( 1-\beta ^{n_y} \right) / \left( 1-\beta \right)$, where $n_y$ is the number of samples for class *y* and $\beta \in \left[ 0,1 \right]$ is a hyper-parameter that controls the growth rate. This re-weighting schema can be applied to any loss $\mathscr {L}(\textbf{p},y)$, leading to a *class-balanced* version $CB(\textbf{p},y)$ that can be expressed as:

1 $$\begin{aligned} CB(\textbf{p},y)=\alpha _{i}\mathscr {L}(\textbf{p},y)=\frac{1}{E_{n_{y}}}\mathscr {L}(\textbf{p},y)=\frac{1-\beta }{1-\beta ^{n_{y}}}\mathscr {L}(\textbf{p},y) \end{aligned}$$

where $y \in \{1, 2,\ldots , C\}$ is the label and $\textbf{p}=[p_1, p_2,\ldots , p_C]$ is the class probability vector estimated by the model. The class weight $\alpha _{i}\propto \frac{1}{E_{n_{y}}}$ is proportional to the inverse of the effective number of samples for class *i*. Choosing $\beta =0$ corresponds to no re-weighting, whereas choosing $\beta =1$ corresponds to re-weighting by the inverse of the class frequency.

Finally, the focal loss is combined with the above weighting scheme to obtain the *class-balanced focal loss*:

2 $$\begin{aligned} CB_{focal}(\textbf{p},y)=- \frac{1-\beta }{1-\beta ^{n_{y}}}\sum _{i=1}^{C}\left( 1-p_{i}^{t} \right) ^{\gamma }log\left( p_{i}^{t} \right) \end{aligned}$$

where $p_{i}^{t} = \text {sigmoid}(z_{i}^{t})$ is the model estimated probability for class *i* and

3 $$\begin{aligned} z_{i}^{t} = {\left\{ \begin{array}{ll} (z_i) &{} \text {if}\, i=y\\ (-z_i) &{} \text {otherwise.}\\ \end{array}\right. } \end{aligned}$$

Parameter $\gamma$ smoothly adjusts the rate at which easy examples are downweighted.

#### Self-supervised pre-training

**Figure 5:**
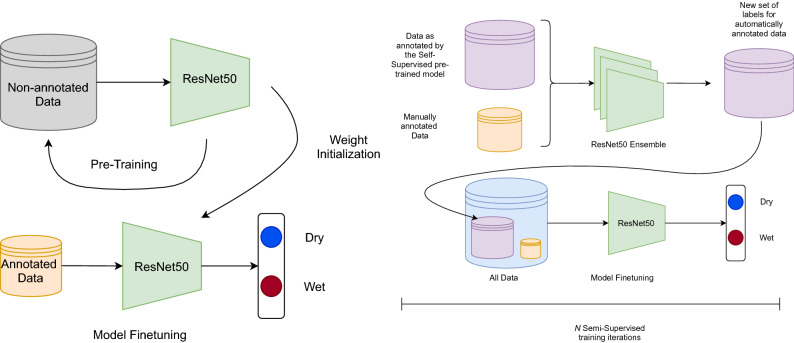
Graphical representations of the Self-supervised pre-training (left) and the Semi-supervised fine-tuning (right).

The network is pre-trained using a self-supervised contrastive technique which leverages the intrinsic similarities present in our dataset, and then fine-tuned to the target classification task, as illustrated in Fig. 5. Specifically, the network is pre-trained in order to learn fine-grained differences in spatio-temporal features by comparing frames taken at different times and at different locations. In our setting, time can be considered as a continuous variable, whereas the camera site is a discrete variable. These two variables are combined to generate sequences including increasingly different samples, inspired by previous works^29^.

Specifically, starting from an anchor sample (A), sequences of five samples *A*, *B*, *C*, *D* and *E* were generated from positive samples with different levels of similarity and one negative sample as reported in Table 3. The degree of similarity was defined based on three factors: the camera site, the date, and the time of the day. In particular, two images were considered similar if they were collected from the same site, if acquired within the same month (30 calendar days, disregarding the year) or if acquired less than 2 h apart. In this way, the concept of seasonality (month) and varying illumination conditions (time of the day) are discretized, and combined with the camera site.

**Table 3:** Similarity between the anchor sample and all other samples in the sequence is determined based on the camera site, date, and time of the day, in that order.

	A	B	C	D	E
Camera site	Anchor	$\checkmark$	$\checkmark$	$\checkmark$	$\times$
Date of the year	Anchor	$\checkmark$	$\checkmark$	$\times$	Don’t care
Time of the day	Anchor	$\checkmark$	$\times$	Don’t care	Don’t care

A $\checkmark$ indicates that the sample is similar to the anchor for the considered factor, whereas an $\times$ indicates that the two samples differ.

A five-stream Siamese network, with a ResNet50 backbone, was used for pre-training. All feature vectors were normalized to unit norm before computing the Euclidean distance. A contrastive loss based on margins was defined as in Yan et al.^29^:

4 $$\begin{aligned} \begin{aligned} \mathscr {L}_{sequence}&={}\max (0,d^2_{AB} - d^2_{AC} + m_1) \\&\quad +\max (0,d^2_{AC} - d^2_{AD} + m_2) \\&\quad +\max (0,d^2_{AD} - d^2_{AE} + m_3) \end{aligned} \end{aligned}$$

where $d^2_{ij}$ is the squared Euclidean distance in feature space between frames *i* and *j*, and $m_i$ represents the distance margin applied to each sample feature representation satisfying the condition $m_3> m_2> m_1 > 0$. The loss aims at pushing sample *B* close to the anchor *A* in feature space, while pushing samples *C*, *D* and *E* further away in this specific order. Since each batch contains *S* sequences, the final loss was $\mathscr {L}= \frac{1}{2S}\sum ^S_{s=1}\mathscr {L}_{s}$. The self-supervised pre-trained backbone was then used to initialize the baseline in lieu of the standard ImageNet pre-training^46^.

#### Semi-supervised training

The proposed solution is based on the well-known SSL *wrapper* technique, also known as pseudo-labelling^47^. Briefly, pseudo-labels are generated for unlabelled frames exploiting an early version of the classifier, which is then refined in an iterative fashion. Class labels produced by multiple classifiers can be further combined to reducing the impact of noisy pseudo-labels. In particular, we considered an ensemble of three different networks with the same architecture (Resnet50) and weights initialized either through self-supervised pre-training, or through previous iterations of self-supervised learning. The predictions were binarized by selecting a threshold corresponding to a $10\%$ false positive rate. Then, the predicted labels from different classifiers were combined using majority voting, and further refined by applying majority voting along the temporal axis to ensure temporal consistency.

Pseudo-labelled training samples were weighted by a factor $\varepsilon$, which is initially set to a low value and then increased at each SSL cycle as the performance of the classifier increases. This hyper-parameter adjusts the trade-off between labeled and unlabeled data, and we experimentally found it was critical to reduce the impact of noisy labels in early iterations^48,49^.

### Temporal (convLSTM) model and training

**Figure 6:**
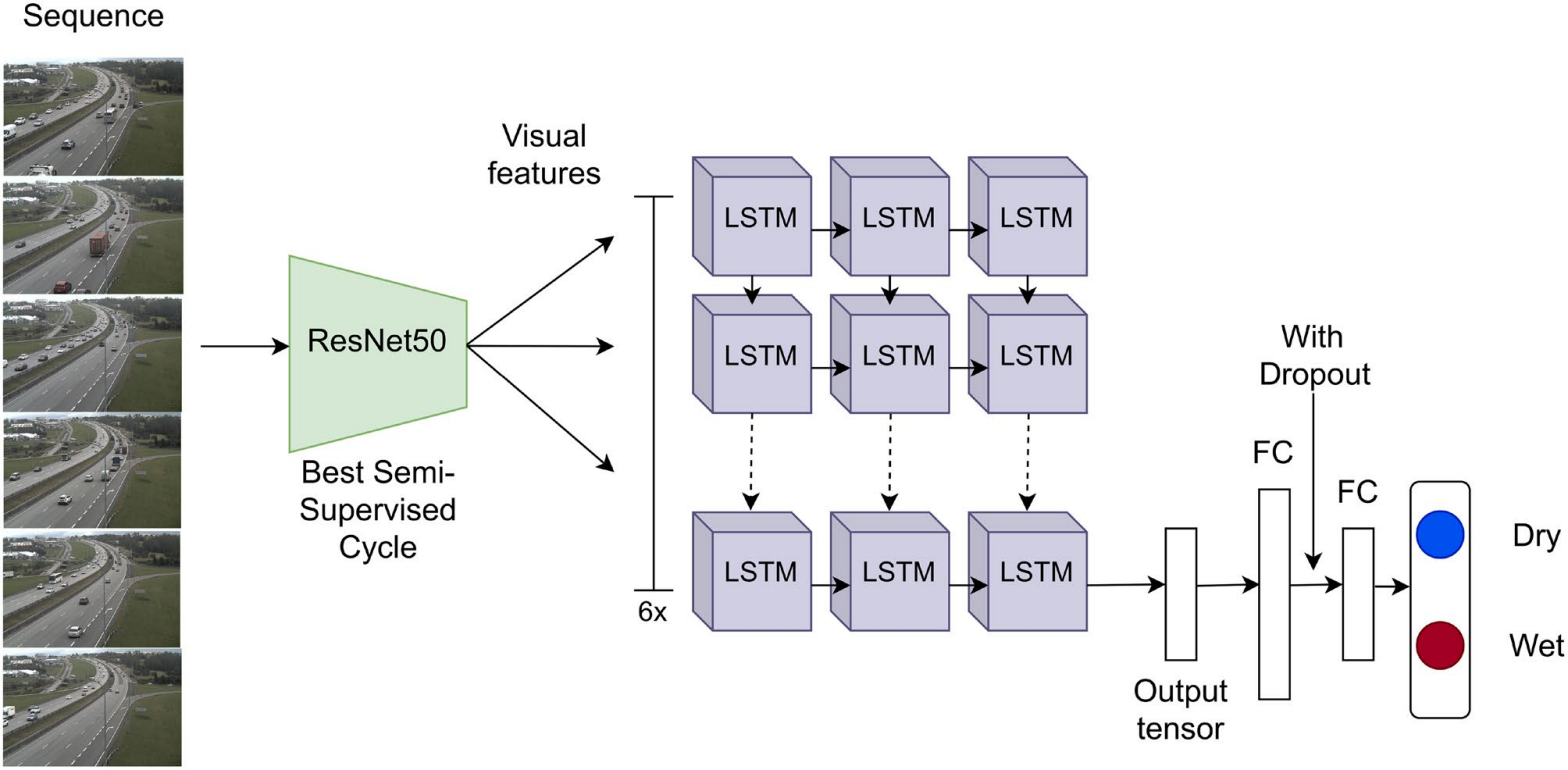
Temporal (convLSTM) model. The architecture is shown in the unrolled form (backpropagation through time).

The temporal model classifies the *i*th frames by looking back to the previous $N-1$ frames. The temporal model consists in a convLSTM model^50^, with a ResNet50 backbone as visual encoder, followed by a stack of three LSTM cells and two fully connected (FC) layers, as depicted in Fig. 6.

We experimented with different variants of the LSTM model. The first variant is the DenseLSTM^51^ in which, similarly to the DenseNet architecture, each of the three LSTMs receives as input the visual features concatenated with the output of all previous cells. In the second variant, the LSTM is substituted by a simple 1D CNN layer, with kernel size equal to 2 and stride 1; the output of the 1D CNN layer is flattened and passed to the two FC layers. In all models, dropout after the first FC layer is used to reduce overfitting.

Temporal sequences were extracted from the continuous data stream using a sliding window approach, characterized by three parameters: the length of the sequence *N*, the sampling rate *s* and the stride *K*. In our experiments, we set $s=1.3$ (1 frame every 3 min) and $N=6$, thus resulting in a time window of 18 min. This time window was selected based on domain knowledge and some preliminary experiments, and is sufficient to capture the evolution of the phenomenon of interest. We further set the stride *K* equal to the sampling rate, for simplicity.

To reduce the computational effort for training, the temporal model is initialized from the by-frame model by means of a two-step transfer learning strategy. In the first phase (*warm-up*), the feature extractor is frozen and only the LSTM is trained, whereas in the second phase also the backbone is fine-tuned. The class-balanced focal loss, introduced in Section “CNN model and training”, is used.

## Experimental settings

### CNN model

All experiments were performed resizing Full HD frames to $480\times 270$ pixels. This preprocessing slightly reduces the classification accuracy, but substantially speeds up training and experiments. Unless otherwise noted, the hyper-parameters for the focal loss were set to $\beta =0.9999$ and $\Gamma =2$ for all experiments. Frames labelled other than wet and dry (i.e., over-exposed, dark, offline, and poor visibility) were removed from the dataset, as explained in Section “Dataset description”. The learning rate is optimized on the training set through the learning rate finder methodology^52^ and set to $1\textrm{e}{-6}$. The Adam optimizer was used for all experiments with a batch size of 16.

The baseline was pre-trained using self-supervised learning on 308,523 sequences for 20 epochs. Each sample of the original training set acted as an anchor *A* in the sequences dataset, whereas samples *B*, *C*, *D* and *E* were randomly picked among the ones satisfying the requirements for the given anchor. The generated sequences were finally split by randomly picking 2982 sequences to be used as validation set, leaving the remaining 305,541 sequences for training. The hyper-parameters of the loss function were set to $m_1=0.2$, $m_2=0.3$ and $m_3=0.4$ (see Section “Methodology” for more details on the loss function) with batch size $S=3$. After the pre-training phase, the backbone was fine-tuned to the desired classification task: the learning rate was optimized through the learning rate finder methodology^52^ and set to $5\textrm{e}{-6}$.

For SSL iterations, pseudo-labels were downweighted by a factor $\varepsilon$, set initially to 0.2 for the first iteration to a maximum of 0.9 for the fourth (and last) SSL iteration.

It is worth noticing that, for the self-supervised pre-training and the four initial SSL rounds, only the data from sites A1–A10 was used, as these sites cover a much wider temporal range then sites B1–B15. However, the latter include a wider variety of scenes and more challenging illumination conditions, and thus are particularly interesting for the temporal model training and evaluation.

### ConvLSTM model

The temporal convLSTM model baseline was trained following a two-step procedure. The optimizer (Adam) and loss (focal loss with $\beta = 0.9999$ and $\gamma = 2$) were the same as for the CNN model. In the warm-up phase, the network is trained for 4 epochs with batch size 64 and learning rate $9\times 10^{-5}$. In the second phase, the whole network is fine-tuned for additional 6 epochs using a lower learning rate $9\times 10^{-6}$ and a batch size of 16. In both SSL rounds, $\varepsilon$ is set to 0.9. The optimal learning, determined experimentally with the learning rate finder algorithm, was $9\times 10^{-5}$ and $9\times 10^{-6}$ for the first and second SSL rounds, respectively. As for the CNN model, experiments were performed resizing Full HD frames to $480\times 270$ pixels. The convLSTM model was trained on 256,882 sequences of $S=6$ frames, extracted from the Training-50K dataset using the methodology described in Section “Data preprocessing and data augmentation”.

### Experiments

All the experiments were performed on a system equipped with a Intel®Core™ i9-9940X CPU with 32 GB RAM and a NVIDIA Titan RTX GPU (24 GB). The models were implemented in Keras v2.3 and Tensorflow v2 frameworks.

### Performance assessment

We considered two classes of performance metrics, at the *frame* and *event* levels. The first set of metrics assess the ability of the model to independently classify each frame as dry or wet. In this case, the performance was evaluated using the *Receiver Operating Curve (ROC)*, with the *Area under the ROC curve (AUC)* as summary performance measure. 95% confidence intervals (c.i.) for the AUCs were calculated using bootstrap (1000 sampling repetitions, with replacement)^53^, whereas *p* values were calculated using the paired non-parametric DeLong method for comparing correlated ROC curves^54^, with Bonferroni correction for multiple tests. To reduce the number of statistical tests, we tested the differences between the two final models, as well as the difference between each semi-supervised iteration and the baseline model.

The second set of metrics capture how accurately wet road events were detected by the system, how many false alarms were generated and how they were distributed across the day or across sites. First, an event is defined as a series of consecutive frames with the same label. Predictions are thus binarized to generate the predicted events. In particular, to seek a balance between specificity and sensitivity and compare models on fair grounds, the threshold was selected at a fixed false positive rate (by-frame) of $10\%$.

Then, the ground truth and detected events were matched along the temporal axis using the *temporal Intersection over Union (IoU)* metric^55^. Specifically, an event was considered detected if the IoU exceeded the 0.2 threshold. Any predicted event that is not associated to a ground truth event was considered a *false positive* or *false alarm*. However, if a predicted event is completely included in a ground truth event but it is not sufficient to reach a IoU greater than the threshold, it is not counted as a false positive nor a true positive detection.

Finally, per-event performance is estimated through a variant of the ROC curve (*Free-Response ROC curve* or *FROC*), which plots the recall vs. the average FP rate. The average FP rate is obtained by dividing the total number of FPs normalized by the camera days, and corresponds to the average number of FPs generated daily by each site. FPs with duration equal to one frame are discarded. To calculate the curve, each detected event is assigned a score equal to the maximum score assigned by the model to the frames included in the event itself. By construction, each event will have a score equal or higher to the threshold at which predictions are binarized.

## Results

### CNN model: self- and semi-supervised pre-training

First, we evaluated the ability of the CNN model to discriminate dry versus wet frames. For this initial set of experiments, sites B1–B15 were excluded, as unlabelled data was not available and the acquisition period was relatively short compared to sites A1–A10. The validation set includes 208 camera days from eight distinct sites, of which four were never seen during training. The CNN model is trained in consecutive steps as introduced in Section “CNN model and training”. The *Self-sup baseline* refers to the model pre-trained following the proposed self-supervised methodology and then fine-tuned on the labeled portion of the dataset, whereas the *Semi-sup X* model refers to the *X*th SSL round on the complete Training-300K dataset.

**Figure 7:**
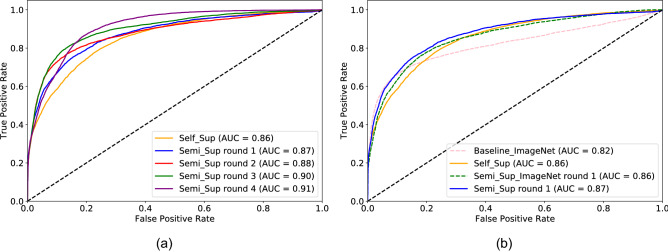
ROC curves of the baseline model with self-supervised initialization (*Self-sup*) and models fine-tuned via SSL on the large Training-300K dataset. (**a**) Performance comparison of four SSL rounds. (**b**) ROC curves of the baseline model with (continuous line) and without (dashed line) *Self-sup* initialization. Models trained with self-supervised initialization achieve higher performance with and without fine-tuning via SSL.

As shown in Fig. 7a, the initial baseline reached a global AUC of 0.855 (0.850–0.860), which increased to 0.910 (0.906–0.913) at the 4th round (*p* $<0.0001$). Interestingly, despite the increasing AUC, only the first three iterations dominate the previous one in ROC space, as demonstrated by the intersecting curves in Fig. 7a. AUC values with 95% c.i. are reported in Table 4. The difference between each *Semi-sup X* model and the *Self-sup* baseline was highly significant (*p* $<0.0001$) on both the validation and test sets.

The performance was very site-dependent, both for the *Self-sup* baseline (per-site AUC: median 0.86, IQR 0.09, range [0.58, 0.97] ) and the final *Semi-sup 4* model (median 0.895, IQR 0.06, range [0.81, 0.98] ). The final performance for sites that were seen/unseen during training was 0.91 (range [0.81, 0.98]) and 0.875 (range [0.84, 0.96] ), respectively. This suggests that the model is not overfitting to the specific sites included in the training set; performance was lower for intrinsically “difficult” sites with challenging weather (e.g., low prevalence of wet events) or acquisition conditions (e.g., over-exposure or cameras located very far from the road).

**Table 4:** AUC and 95% C.I. on the validation and test sets.

Model	Validation set (A1–A10)	Test set
Self_Sup	0.857 (0.852–0.861)	0.890 (0.885–0.895)***
Semi_Sup round 1	0.874 (0.869–0.879)***	0.912 (0.908–0.917)***
Semi_Sup round 2	0.878 (0.872–0.883)***	0.914 (0.909–0.918)***
Semi_Sup round 3	0.904 (0.889–0.908)***	0.923 (0.919–0.928)***
Semi_Sup round 4	0.911 (0.907–0.914)***	0.920 (0.915–0.923)***

Differences between each SSL round and the *Self_sup* baseline were tested for statistical significance. Asterisks (*) indicate *p* value $< 0.05$ (*), *p* value $< 0.01$ (**), and *p* value $< 0.001$ (***), respectively.

An ablation study was conducted in which self-supervised pre-training was removed from the pipeline. In this experiment, the CNN pre-trained on ImageNet was directly fine-tuned on the Training-50K dataset, excluding sites B1–B15 (*Baseline-ImageNet*), and then one SSL round was performed on the Training-300K dataset (*Semi-sup-ImageNet*). The AUCs are reported in Fig. 7b. The *Self-sup* baseline, with AUC equal to 0.855 (0.850–0.860) outperforms the *ImageNet* AUC, 0.819 (0.810–0.826) by a large margin (*p* $<0.0001$). The performance gap is maintained, albeit reduced, after one SSL round, with AUC 0.874 (0.869–0.879) versus 0.859 (0.854–0.864) (*p* $<0.0001$).

### ConvLSTM performance

Then, we move on to evaluate the performance of the convLSTM models, introduced in Section “Temporal (convLSTM) model and training”, fine-tuned from the final model reported in Section “CNN model: self- and semi-supervised pre-training”. As a preliminary experiment, the LSTM, DenseLSTM and 1D CNN models were compared on a subset of the Training-50K dataset, excluding the B1–B15 sites. The AUC obtained with the LSTM, DenseLSTM and 1D CNN was equal to 0.87, 0.88 and 0.85, respectively. For the main experiments, the LSTM model was selected because it provided the best trade-off between computational complexity and accuracy.

**Figure 8:**
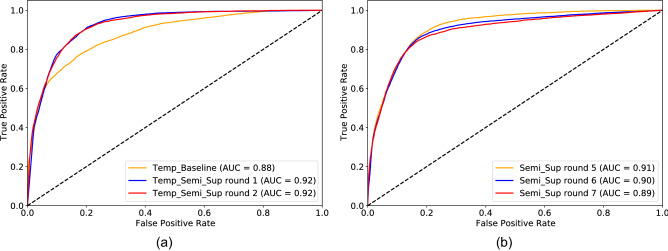
ROC curves of the convLSTM model (**a**) and 2D CNN (**b**) trained on the entire Training-300K dataset. Both models were fine-tuned from the *Semi-sup 4* model (Fig. 7a). Three SSL rounds were repeated for both models.

All subsequent experiments were performed on the complete dataset (including sites B1–B15). The initial training was performed on the labelled Training-50K dataset (*Temp-Baseline*) and then fine-tuned for two additional semi-supervised iterations (*Temp-Semi-Sup X*). To compare the convLSTM and CNN models on equal grounds (same amount of training iterations and same validation sets), the CNN was further fine-tuned for three additional rounds on the complete dataset.

As shown in Fig. 8, the *Temp-Baseline* model achieved an initial AUC of 0.879 (0.875–0.883), which is higher than the previous *Self-sup* baseline, but lower than the best *Semi-sup X* model (*p* $<0.0001$). The performance then increases to 0.924 (0.921–0.927) with two SSL rounds (*p* $<0.0001$), with each iteration outperforming the previous one. The performance of the CNN model peaks at 0.914 (0.920–0.917), then reduces to 0.892 (0.888–0.896). AUC values with 95% c.i. are reported in Table 5. The difference between each *Temp-Semi-sup X* model and the *Temp-Baseline* was highly significant (*p* $<0.0001$) on both the validation and test sets.

**Table 5:** AUC and 95% C.I. on the validation and test sets.

Model	Validation set	Test set
Temp_Baseline	0.879 (0.875–0.883)***	0.890 (0.885–0.895)***
Temp_Semi_Sup round 1	0.923 (0.921–0.926)***	0.907 (0.902–0.912)***
Temp_Semi_Sup round 2	0.924 (0.921–0.927)***	0.923 (0.919–0.928)***
Semi_Sup round 5	0.914 (0.911–0.917)***	0.914 (0.909–0.918)***
Semi_Sup round 6	0.899 (0.895–0.902)***	0.883 (0.876–0.889)***
Semi_Sup round 7	0.892 (0.888–0.896)***	0.886 (0.880–0.892)***

Differences between each SSL round and the *Temp_baseline* were tested for statistical significance. Asterisks (*) indicate *p* value $< 0.05$ (*), *p* value $< 0.01$ (**), and *p* value $< 0.001$ (***), respectively.

**Figure 9:**
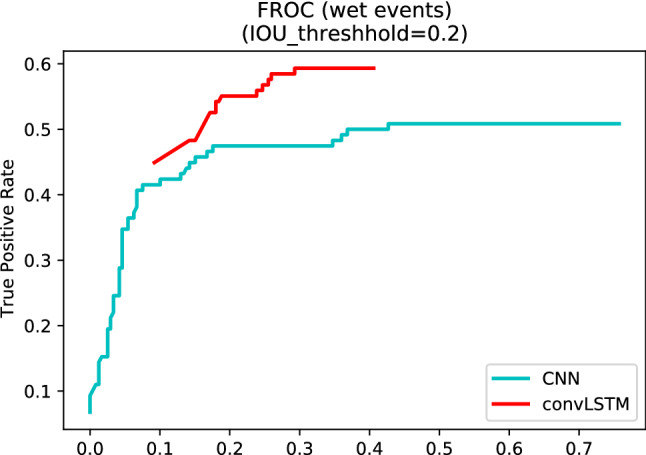
Per-event recall versus average number of FP events.

Notably, the convLSTM and CNN models, although reaching similar performance in terms of AUC, have substantially different behaviors at the event level. For the convLSTM, sensitivity increases from 50% (60/118) to 60% (71/118), whereas the FP rates drops from 0.689 (181/263) to 0.37 (99/263), as detailed in Table 6. Of the 47 false negatives, 33 are completely undetected, whereas the rest fail to reach the IoU threshold. Many of the FP events are generated by conditions in which the road is damp, but not wet, at least according to the more restrictive definition adopted in this work. Specifically, roughly 25% of the FP events occur in the tail of a wet event (13 within a 1 h window and 10 within a 3 h window), and 25% occur in the early morning between 4am and 9am. A similar behavior is observed for the 2D CNN model. The superior performance of the convLSTM model is further highlighted by the FROC curves in Fig. 9.

The average inference time, measured on the validation set on a system equipped with a Intel®Core™i9-9940X CPU with 32 GB RAM and a NVIDIA Titan RTX GPU (24 GB VRAM), was equal to 0.024 seconds/frame for the CNN model, and 0.034 seconds/frame for the ConvLSTM model. Inference time increases since the ConvLSTM model takes as input six frames, all of which need to be processed by the convolutional backbone. However, the time efficiency of the ConvLSTM model can be optimized when executed on a continuous video stream, as adjacent sequences share most of the computation, and thus the extracted features can be cached. It should be noted, however, that the measured inference time does not take into account the latency required to transfer the images to a cloud server with comparable hardware resources. The proposed models would require further optimization and/or compression to be executed at the edge, e.g., by a smart camera.

### Factors affecting performance

A few factors were found experimentally to influence the performance of the convLSTM model. First, the performance is still site-dependent (median 0.915, IQR 0.11, range [0.80, 0.99]), similarly to the the previous *Semi-sup 4* model (median 0.895, IQR 0.06, range [0.81, 0.98]. The final performance for sites that were seen/unseen during training was 0.91 (range [0.80, 0.96]) and 0.915 (range [0.80, 0.99]), respectively, which further confirms the ability to generalize to novel sites.

**Table 6:** Comparison of the best semi-supervised models for the plain CNN and the temporal convLSTM model.

	Recall	FP Events	FN Events	TP Events	10% FP Rate Threshold
Best CNN	0.50	181	58	60	0.90
Best convLSTM	0.60	99	47	71	0.96

Second, the frames were split by time of acquisition, and time-specific AUCs were calculated for each hour of the day (Table 7). Since sunrise and sunset vary across different seasons and sites, frames before 8am and after 5pm were pooled. Performance drops in the early morning, when the road is often damp, and late afternoon/evening, mostly due to the more challenging illumination conditions.

**Table 7:** Performance (AUC) measured on frames acquired at different hours of the day for the convLSTM model.

Time range	ROC-AUC
03:00–08:00	0.897
08:00–09:00	0.900
09:00–10:00	0.923
10:00–11:00	0.933
11:00–12:00	0.952
12:00–13:00	0.949
13:00–14:00	0.952
14:00–15:00	0.929
15:00–16:00	0.893
16:00–17:00	0.894
17:00–23:00	0.952

**Figure 10:**
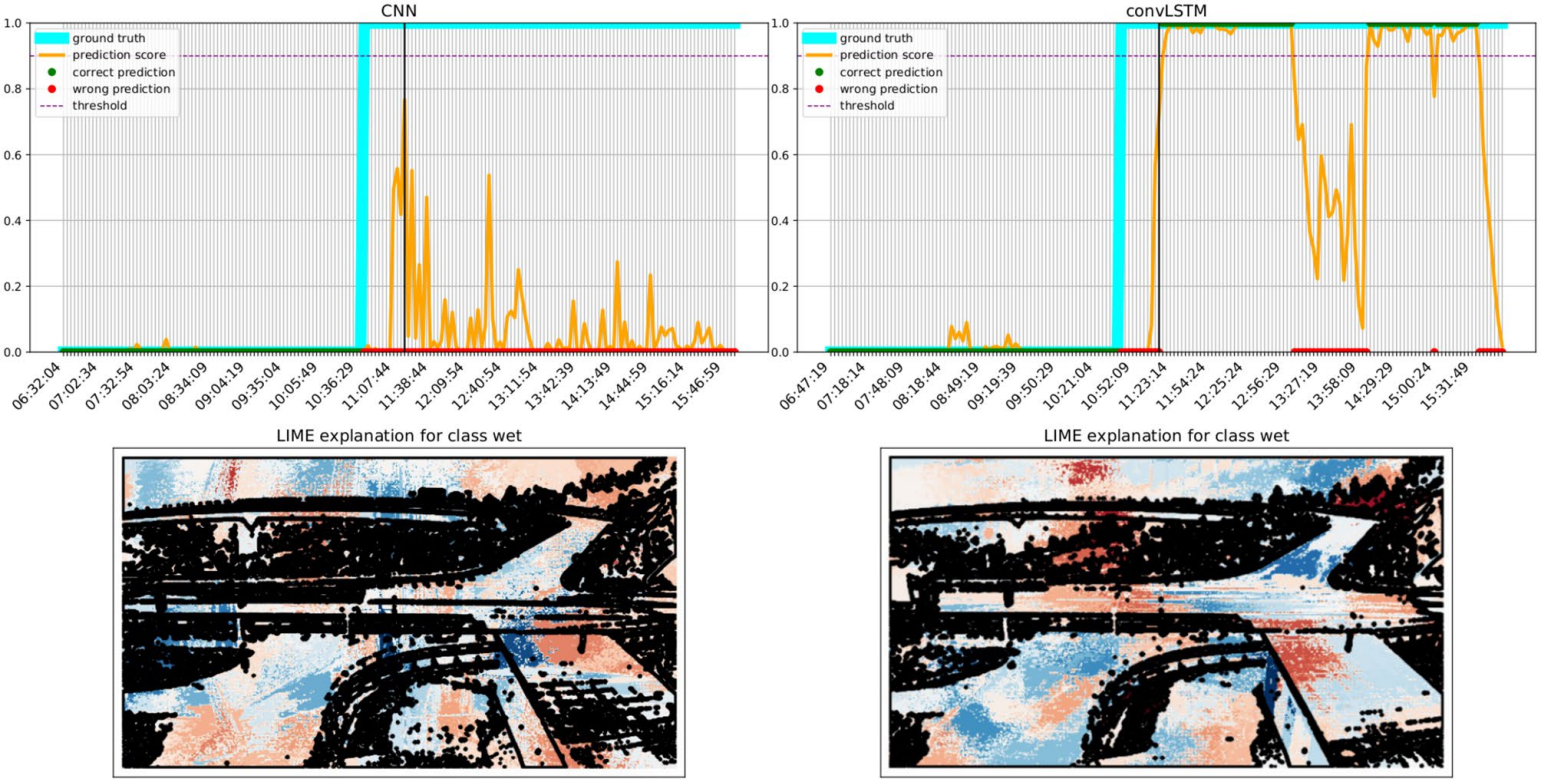
LIME explanation over a transition frame as predicted by both the models. Blue areas push the model towards the wet class, whereas red areas push towards the dry one. Best viewed in color.

**Figure 11:**
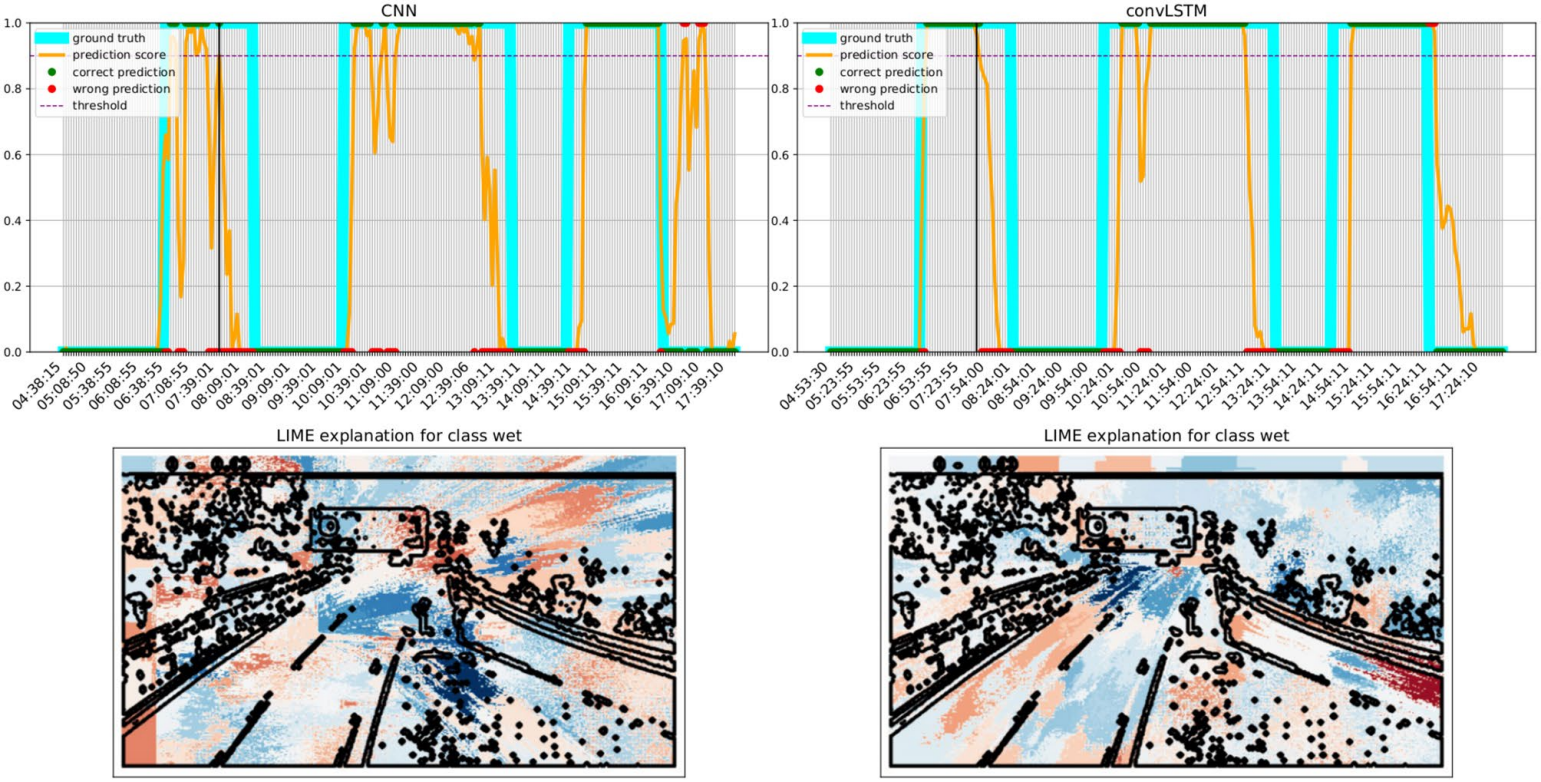
Comparison of the transition profile for a severe event. The convLSTM model especially improves the detection of the two events in the morning. Blue areas push the model towards the wet class, whereas red areas push towards the dry one. Best viewed in color.

**Figure 12:**
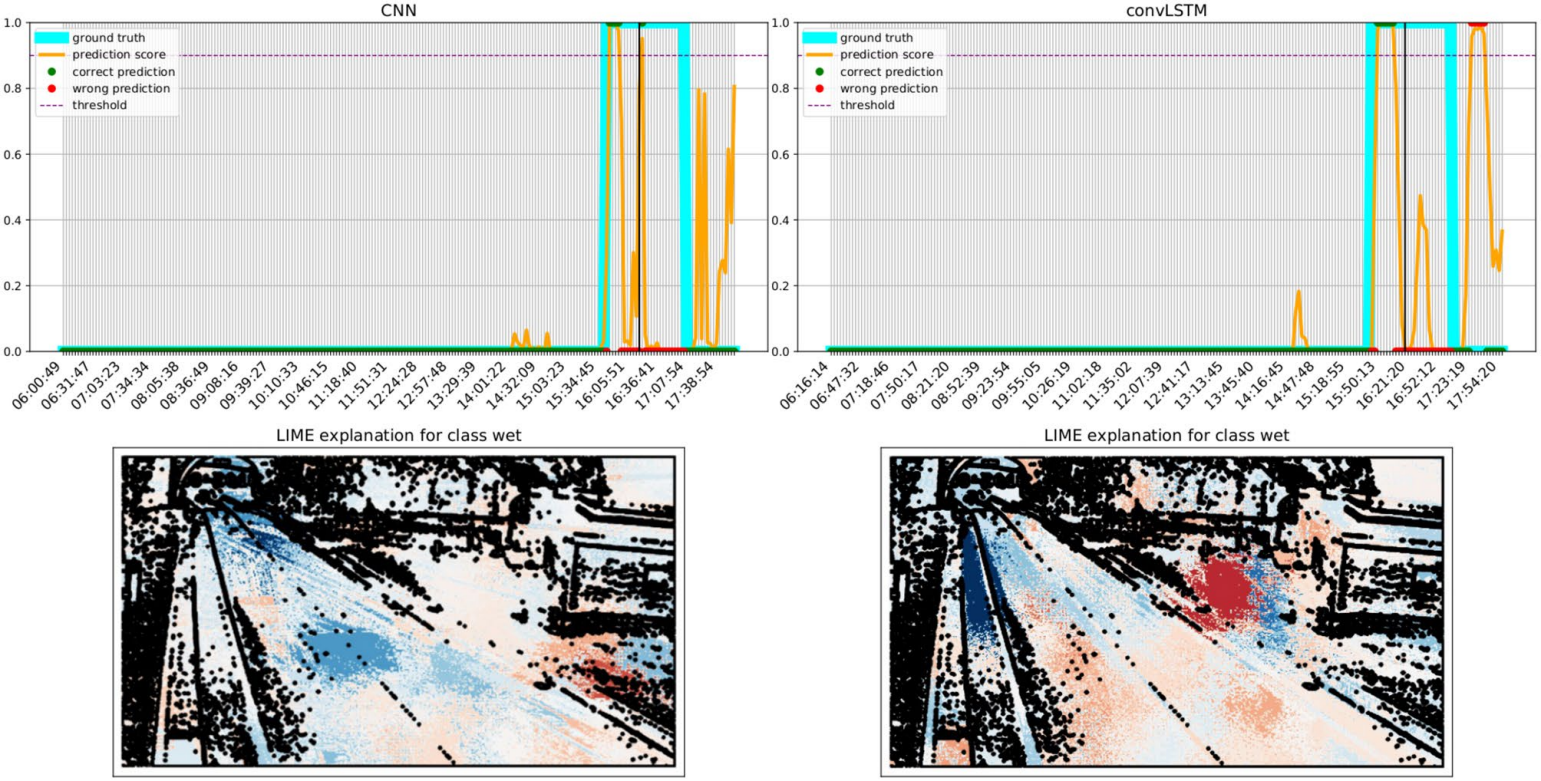
LIME explanation over a transition frame as predicted by both the the models. Blue areas push the model towards the wet class, whereas red areas push towards the dry one. Best viewed in color.

### Transition plots and explainability

We finally investigate the difference between the best CNN and convLSTM models in the form of transition plots. A transition plot shows how the score assigned by the model to each frame evolves during a wet event, and thus how confident and consistent the model predictions are. On the same plots we also report the ground truth value for each frame, and the threshold used to trigger a wet frame prediction (as a dotted line). LIME^56^ was used on a representative frame (identified by a black line) to highlight how different regions contribute to the predictions.

Transition plots shown in Figs. 10, 11 and 12 were calculated for three severe events occurring at sites with very different morphology. Even when both models technically detect the event, the predictions of the convLSTM are a more faithful representation of the underlying phenomenon. LIME plots show that the ConvLSTM model is also more capable of distinguishing which parts of the road are indeed wet: for instance, it can detect the presence of water in the tunnel at the end of the road in Fig. 12. Notably, the parts highlighted by the convLSTM model were found to be more similar to those used by the raters when labelling the images. Both models also rely on contextual cues, such as the presence of fog and humidity between the trees in Fig. 10. As a matter of fact, we noticed a significant drop in performance when the model was trained only on the segmented road stretch (results not shown for brevity).

## Discussion

The results reported in this paper highlight the effectiveness of self-supervised semi-supervised learning to leverage partially labelled datasets for road condition estimation: this is in line with previous research in weather analysis^6^, as well as in the general computer vision domain^27^. In the absence of a public benchmark, it is not possible to directly compare our methodology with previous studies^6,13,14^. Nonetheless, several interesting conclusions emerge.

First of all, we adopt a stricter definition of “wet” road than previous literature^6^. This decision is motivated by the most common use cases in road safety and infrastructural monitoring, specifically, the need to reduce false alarms and thus identify only those instances which pose a significant threat to road safety. This definition has an impact on both training and evaluation, as we noticed that many FPs originate from situations in which the road is damp, but not “wet”.

We observed that hand-crafted labels are, to a certain degree, subjective. We counterbalanced this potential limitation with consensus meetings to discuss borderline cases. Precisely identifying the start and end of each event, however, still remains challenging, as the transition between “dry” and “wet” is often smooth. In future work, we plan to incorporate temporal label smoothing to account for the uncertainty in the exact start and end time of each event^57^.

To the best of our knowledge, this is the first study that accounts for the intra-frame correlation both at training and test time, thus reporting performance at the frame and event level, for road condition estimation and weather analysis. To this aim, novel strategies for self-supervised pre-training and temporally-aware data augmentation were proposed. The proposed convLSTM model outperforms CNNs and achieves better qualitative results, as evidenced by explanations computed using the LIME technique. However, the convolutional backbone is pre-trained on individual frames using semi- and self-supervised techniques. This choice was motivated by several practical motivations: the convLSTM training time is much higher than the CNN one (three-four fold increase), and curriculum learning (i.e., training schedules in which the network is exposed to increasingly complex tasks) was found to stabilize LSTM training^51^. In addition, it becomes possible to pre-train on sites for which video sequences are not available. On the other hand, training directly the temporal model may converge to a different, or even better, solution.

Another limitation of the present study is that the impact of self-supervised pre-training on the final performance could be better explored, possibly extending the self-supervised technique to model temporal sequences, or comparing against different self-supervised pre-training techniques like clustering-based ones^26^.

Finally, while our focus was mostly on comparing the relative performance of different models, it should be noticed that the absolute performance could benefit from increasing both temporal and spatial resolution. To test this hypothesis, the final convLSTM model was fine-tuned for six additional epochs after doubling the image size from $480 \times 270$ to $960 \times 560$. The overall AUC increased from 0.92 to 0.93; the per-site AUC increased for five sites (average increase 0.046) and decreased for three sites (average decrease 0.017), depending on the distance between the camera and the road. It should also be noticed that all models were trained on grayscale images, due to the limited availability of RGB videos, which is also likely to have a negative impact. Nonetheless, since the focus of our study is on the relative performance of different models when compared on the same data, the conclusions should not be affected by these minor shortcomings.

## Conclusions and future work

In this work, we presented a novel approach for road condition estimation, and particularly the detection of wet road events in untrimmed video sequences. While most previous studies in the literature employ standard CNNs for frame-by-frame classification, we exploit the temporal correlation between adjacent frames by leveraging a convLSTM model. The adopted training strategy combines novel self-supervised pretraining strategies with a straightforward pseudo-labelling approach to exploit a large pool of unlabelled data.

Experimental results highlight how the performance of the convLSTM improves over a standard CNN, not only detecting more events with lower FP rates, but yielding predictions which are more consistent with human perception. The temporal model appears to generalize well to sites that were not seen during training, and the differences appear to be related to the intrinsic difficulties, e.g., specific illumination or weather conditions associated to a given site.

Future work will focus on improving experimental results, e.g., by expanding self-supervised pre-training to handle sequences instead of individual frames. Additional challenges to be tackled involve distinguishing borderline conditions from potential labelling noise due to inter-rater variability, as well as misclassification in the pseudo-labelling iterations. It is possible that manually labelling a larger portion of the dataset could further improve performance. To reduce as much as possible the labelling costs, more robust and principled SSL strategies could help distinguishing difficult samples from noisy ones, and avoid the need to tune the additional hyper-parameter $\varepsilon$. Further improvements could be achieved by evaluating alternative architectures for the temporal model, including those based on 3D convolutions^58^.

Finally, even though the classification problem is technically binary, problems related to long-tailed distributions occur as certain weather and/or road conditions are intrinsically rare and occur only in a limited number of sites. In these cases, fine-tuning the model to individual sites may increase performance, although collecting larger datasets could alleviate this issue in the future.

## Supplementary information

Supplementary Information.

## Data Availability

The datasets generated and/or analyzed during the current study are not publicly available due to licensing and privacy constraints. Data are however available from the corresponding author upon reasonable request and with permission from Waterview srl.
