# Supplementary material for: Self-supervised and semi-supervised learning for road condition estimation from distributed road-side cameras
Source: Sci Rep. 2022 Dec 26;12:22341. doi: 10.1038/s41598-022-26180-4 (PMC9792450; doi:10.1038/s41598-022-26180-4)
Supplement: Supplementary file 1 — Supplementary Information. [file 41598_2022_26180_MOESM1_ESM.pdf]

Appendix

Camera site	Acquisition period	Orientation	Camera model
A1	02/2020 - 05/2020	-45	AXIS_P1435-LE
A2	02/2020 - 05/2020	45	AXIS_P1435-LE
A3	07/2018 - 07/2019	-90	AXIS_Q1765
A4	10/2018 - 07/2019	180	HIKVISION_DS-2CD4525FWD-IZ (H)
A5	10/2018 - 05/2020	180	AXIS_P1435-LE
A6	10/2018 - 07/2019	45	HIKVISION_DS-2CD4525FWD-IZ (H)
A7	07/2018 - 09/2018	45	HIKVISION_DS-2CD4525FWD-IZ (H)
A8	10/2019 - 11/2019	180	HIKVISION_DS-2CD4525FWD-IZ (H)
A9	08/2018 - 07/2019	0	AXIS_Q6054-E
A10	02/2020 - 05/2020	45	AXIS_P1435-LE

Table 8. Characteristics of the cameras included in this analysis. Orientation denotes the direction the camera is pointed towards, calculated with respect to the North. Information was not available for all sites.
